# Initial Results from SQUID Sensor: Analysis and Modeling for the ELF/VLF Atmospheric Noise

**DOI:** 10.3390/s17020371

**Published:** 2017-02-14

**Authors:** Huan Hao, Huali Wang, Liang Chen, Jun Wu, Longqing Qiu, Liangliang Rong

**Affiliations:** 1 College of Communications Engineering, PLA University of Science and Technology, Nanjing 210007, China; whhaohuan@163.com (H.H.); liang_ch@sina.com (L.C.); 2 Shanghai Institute of Microsystem and Information Technology, Chinese Academy of Sciences, Shanghai 200050, China; wujun@mail.sim.ac.cn (J.W.); lq.qiu@mail.sim.ac.cn (L.Q.); rong_elec@mail.sim.ac.cn (L.R.)

**Keywords:** amplitude probability density, atmospheric noise, superconducting quantum interference device, S*α*S distribution

## Abstract

In this paper, the amplitude probability density (APD) of the wideband extremely low frequency (ELF) and very low frequency (VLF) atmospheric noise is studied. The electromagnetic signals from the atmosphere, referred to herein as atmospheric noise, was recorded by a mobile low-temperature superconducting quantum interference device (SQUID) receiver under magnetically unshielded conditions. In order to eliminate the adverse effect brought by the geomagnetic activities and powerline, the measured field data was preprocessed to suppress the baseline wandering and harmonics by symmetric wavelet transform and least square methods firstly. Then statistical analysis was performed for the atmospheric noise on different time and frequency scales. Finally, the wideband ELF/VLF atmospheric noise was analyzed and modeled separately. Experimental results show that, Gaussian model is appropriate to depict preprocessed ELF atmospheric noise by a hole puncher operator. While for VLF atmospheric noise, symmetric *α*-stable (S*α*S) distribution is more accurate to fit the heavy-tail of the envelope probability density function (pdf).

## 1. Introduction

Extremely low frequency (ELF, defined here as 300–3000 Hz) and very low frequency (VLF, defined here as 3–30 kHz) radio waves have efficient long-range propagation (attenuation rates typically a few dB/Mm at VLF and much smaller, of the order of a few tenths dB/Mm at ELF) in the so-called earth-ionosphere waveguide [1] and comparatively deep penetration into conducting medium such as Earth and seawater. Consequently, they have wide applications ranging from naval communication [2] and geophysical prospecting [3] to underground communication [4].

In order to radiate electromagnetic waves, the dimensions of antenna need to be of the order of the wavelength of the radiation to have an efficiency of any practical significance. Many naval transmitters use tuning elements to force the the antenna to resonate at the working frequency to compensate the shortage of the antenna size. However, the tuning elements provide reasonable efficiency and sized antenna of the transmitter at the expense of a very small resonate bandwidth of the antenna. Consequently, traditional analysis and modeling for ELF/VLF noise are concentrated on narrow-band characteristics (typical bandwidth is 5% of the center frequency). A detailed description of this work can be found in [5]. Recently, ionosphere heating has provided a new way to generate wideband ELF/VLF communication signals, which can raise the communication rate significantly [6]. Experimental results show that the communication bandwidth at 1.51 kHz reaches as high as 400 Hz [7], which encourage us to study the wideband characteristics of the ELF/VLF noise. Mixed Gaussian distribution and Hall model are adopted to describe the probability density function (pdf) of the wideband ELF and VLF noise, respectively in [7]. However, the analysis is limited in specific data and the fitting performance of the model need more sufficient comparison with other widely used models.

Magnetic field sensors are preferred for receiver instead of electric field sensors because they can provide superior noise response at the low end of the frequency range [8]. Nowadays, available magnetic field receivers are fluxgates [9], total field magnetometers [10], induction coils [11], and others [12]. They all have their pros and cons. Fluxgates are not sensitive enough for the application of communication. However, total field magnetometers are not fast enough to provide efficient communication. For the induction coil, two different types of magnetic antennas are constructed, i.e., with or without ferrite core at the center. Although antennas with ferrite core have smaller size, it is a concern that the sensitivity will change with temperature and strong fields can cause nonlinear response [8]. On the other hand, the size of antenna with air core is very large. The gain of traditional magnetic receiver is frequency-varying and the calibration need to inject a series of known-amplitude signals at the input of the preamplifier. Even for the Atmospheric Weather Electromagnetic System for Observation, Modeling, and Education (AWESOME) receiver including an internal calibration circuit which generates a pseudorandom digital sequence 1023 b long, with bit frequency of ∼256 kHz, the amplitude response of the receiver is only about ∼250 Hz and up to 200 individual frequency components are processed separately [13]. Furthermore, the sensitivity of the magnetic antenna is limited by the bandwidth of the signal, i.e., it behaves poorer for receiving wideband signals. The 3-dB cutoff frequencies of AWESOME are at about 800 Hz where the line transformer begins to attenuate the signal, and at 47 kHz where the antialiasing-filter cutoff lies. The noise floor of the AWESOME receiver with 200 Hz bandwidth over the VLF range (18∼30 kHz) is about 4∼5 fT [13]. However, the noise floor of the receiver with the same bandwidth over the ELF/VLF range of interest (1.5∼4.5 kHz) will increase significantly.

Direct current (dc) Superconducting QUantum Interference Device (SQUID), consisting of dual Josephson junctions connected in parallel to form a loop, is very sensitive to the magnetic flux threading the loop [14]. Moreover, the SQUID sensor can be fabricated with a size about 1 cm^2^ [15], which is more suitable to be the antenna of a mobile receiver compared with the traditional ones. The gain of the receiver within the frequency range of interest is independent of frequency and thus it can be easily calibrated with a known-amplitude signal in the center of a magnetically shielded room (MSR). The most significant advantage of the application of the SQUID is that they allow the compact construction of three-axis receivers. The motion-related noise are mainly caused by the effect of sensor rotation in the Earth’s magnetic field. As the field is uniform on the physical scale of SQUID, the Earth’s field component can be removed as a dc component by summarizing the vector components [2,16]. The first ELF reception experiment by a low-temperature (4.2 K) SQUID was reported for submarine communication by [2]. A more detailed description of the low-temperature SQUID based receiver was given in [16]. Similar low frequency mobile receivers using SQUID can be also found in [17,18].

Existing applications of SQUID sensor are concentrated on the reduction of atmospheric noise [19,20,21]. However, the analysis and modeling for the wideband ELF/VLF noise are rarely reported in state-of-art of works. The types of modulations and the corresponding optimal detector are highly dependent on the characteristics of the communication channel. As a result, the SQUID based optimal receiver cannot be derived for the wideband communication using ionosphere heating. In this paper, we adopted a low-temperature SQUID receiver to observe ELF/VLF atmospheric noise during seven days. The field data are preprocessed to suppress baseline wandering and harmonics by symmetric wavelet transform and least square (LS) estimation before further analysis. Statistical and fitting experiments are performed for the processed data to investigate the wideband model of the ELF/VLF noise and initial results are derived in this paper.

## 2. System Description

**Figure 1:**
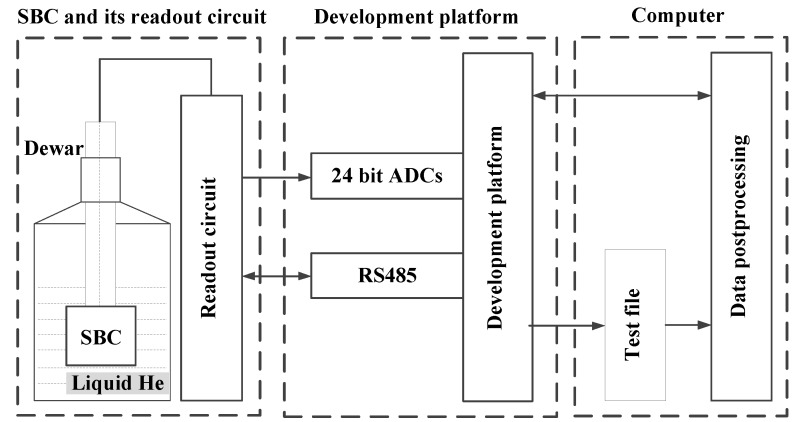
The diagram of the low-temperature superconducting quantum interference device (SQUID) receiver.

As SQUIDs deploying low-transition temeprature superconductors (LTS) can provide a lower magnetic flux noise floor and the corresponding technology is much more refined than high-temperature ones, we adopt the LTS sensor as the antenna of the ELF/VLF receiver. The LTS receiver used in our experiment was fabricated by the Shanghai Institute of Microsystem and Information Technology (SIMIT), Chinese Academy of Sciences [22]. A block diagram of the receiver is shown in Figure 1.

**Figure 2:**
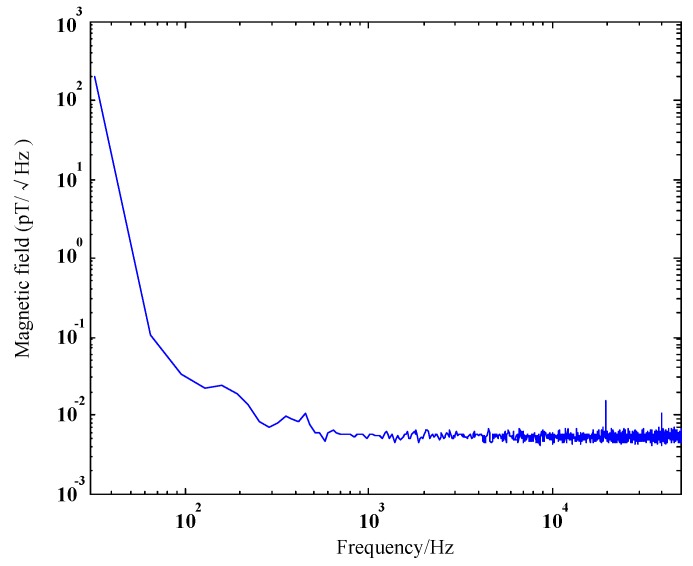
Magnetic noise spectra of the mobile SQUID system measured inside a magnetically shielded room (MSR).

The system consists of three components, i.e., SQUID bootstrap circuit (SBC) and its readout circuit, development platform and computer. The SBC is a new dc SQUID readout circuit operating in the voltage bias mode [22], which can effectively reduce the preamplifier noise below the intrinsic noise of SQUID sensor. More details about the SBC in our experiment can be found in [22]. The development platform is based on CompactRIO developed by National Instruments, which has excellent adaptability in field environment. The high-precision data are sampled at a 100 kHz sampling rate by 24 bit analog-to-digital converters (ADC). The digital data are then stored in the computer and some postprocessing methods listed in later sections are performed on them. The noise floor of the SQUID sensor in the center of a medium magnetically shielded room measured by a spectrum analyzer (Agilen 35670A) is shown in Figure 2 and the noise is about 6 fT/$\sqrt{\mathrm{Hz}}$@1.51 kHz. The noise below 100 Hz is very large because the measurement was conducted at day time with the influence of some external disturbances like a subway nearby. The large low frequency noise presented in Figure 2 should be mostly contributed from the urban environment noise since it differs much at daytime and at midnight. At midnight, we can find that the corner frequency of the 1/f noise is about 3 Hz and the low-frequency components are much smaller than that in Figure 2.

## 3. Data Preprocessing

A continuous observation during seven days for ELF/VLF atmospheric noise was conducted at Hoxtolgay, China in March, 2014. As the vertical magnetic field component is usually much smaller than the horizontal ones near the ground [13], only a single SQUID sensor was chosen for this investigation. It was placed in a dewar made of fiberglass full of liquid helium at a temperature of 4.2 K and the atmospheric noise in the south-north direction in the horizontal plane was recorded during 08:00∼11:00 UT from 2nd to 7th March. The cryostat was half flush-mounted in the Earth and the wind was shielded. The atmospheric noise during this time were quite low. Limited by the storage ability, the data were sampled at a 100 kHz sampling rate and only stored twice an hour with a length of 20 s each time. The measurement site is far from cities but with a power transmission line about 5 km away. As the measurement was conducted under magnetically unshielded environment, the measured data suffered from baseline wandering caused by geomagnetic activity (daily variation and vibration may also cause baseline wandering) and harmonics radiated from the power line nearby. In order to get an accurate analysis and modeling for the ELF/VLF atmospheric noise, the baseline wandering and the harmonics has to be eliminated before further data processing.

### 3.1. Baseline Correction

**Figure 3:**
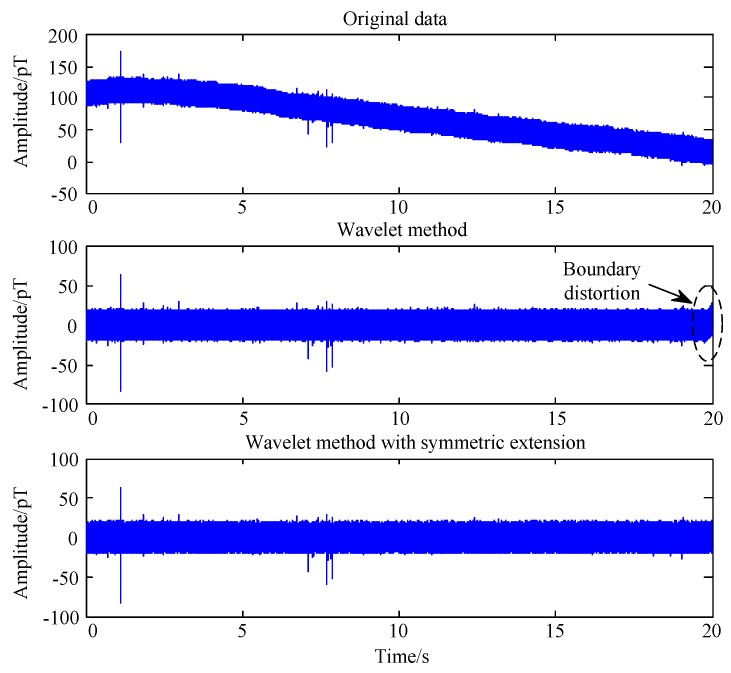
Comparison of one section of 20 ms length of the time record after baseline calibration.

Baseline wandering was often observed in low-frequency measurement [19,23], which would obscure and even deteriorates the analysis result. Consequently, it is necessary to correct the baseline of the observations to extract buried signals [24]. As the atmospheric noise often exhibits non-stationary characteristics, wavelet based methods can provide multi-resolution analysis for the measured magnetic field signal. Recently, Bouchedda et al. [19] proposed two noise reduction techniques to remove atmospheric noise from airborne transient electromagnetic data via a stationary wavelet transform. Li et al. [20] adopted a combined wavelet transform method to reduce the white Gaussian noise and baseline drift jointly for the airborne transient electromagnetic data. Furthermore, Wang et al. [21] suppressed baseline drift effectively by using a wavelet-based correction algorithm. Consequently, wavelet transform is adopted to correct baseline drift by choosing appropriate wavelet basis and decomposition level in this paper. The two-scale equations are given by

(1) $$\phi \left(t\right)=\sqrt{2}\sum_{n}h_{0}\left(n\right)\phi \left(2t-n\right),$$

(2) $$\psi \left(t\right)=\sqrt{2}\sum_{n}h_{1}\left(n\right)\phi \left(2t-n\right),$$

where, ϕ(t) is the scaling function, ψ(t) is the wavelet function. The wavelet transform can be regarded as a filter bank and $h_{0}$ and $h_{1}$ are filter coefficients. The baseline wandering can be corrected by discarding the estimated low frequency components decomposed by the wavelet transform. As the length of real world signal is finite, the wavelet denoising method would cause distortion on the boundary of the signal. However, it was ignored in many wavelet-based filtering methods [20,21]. In order to alleviate the adverse boundary effect by traditional wavelet method, we extend the data at both ends of the according time series with a mirror extension. The corresponding waveform processed by wavelet methods, adopting “sym8” base [21] and 14 levels are shown in Figure 3. As the sampling rate is 100 kHz, the frequency components lower than 3 Hz will be filtered. It is shown that wavelet methods can provide perfect baseline correction results for our data. Moreover, the boundary effect caused by traditional wavelet methods can be eliminated by extending the data.

### 3.2. Harmonic Suppression

**Figure 4:**
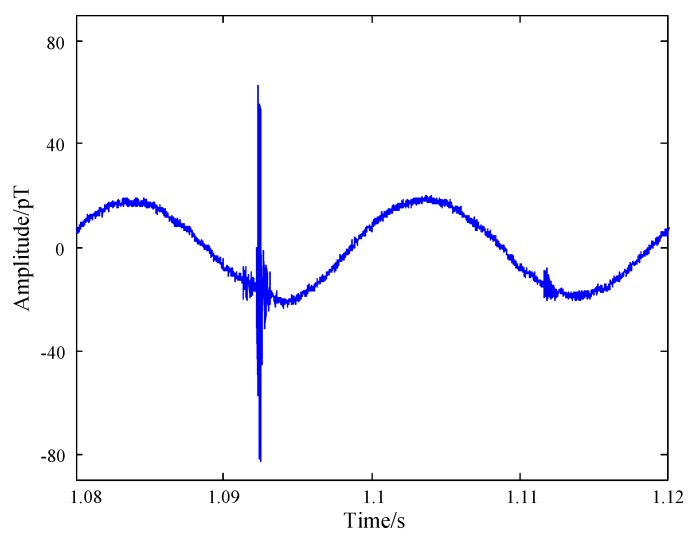
Waveform of the atmospheric noise before harmonic suppression.

It can be verified in Figure 4 that the measured magnetic data suffered from harmonics (fundamental frequency is 50 Hz) with large amplitude up to 20 pT. As the amplitude of harmonics are much larger than the atmospheric noise, it has to be eliminated before further data processing and analysis. The received signal y(t) consists of the atmospheric noise s(t) contaminated with harmonics p(t), given by

(3) y(t)=s(t)+p(t),

where p(t) can be further represented by

(4) $$p\left(t\right)=\underset{X(t)}{\underset{︸}{\left[\begin{array}{c} \cos\left(2\pi f_{0}t\right)\cdots \cos\left(2K\pi f_{0}t\right)\;\sin\left(2\pi f_{0}t\right)\;\cdots \sin\left(2\pi Kf_{0}t\right) \end{array}\right]}}{\underset{P^{T}}{\underset{︸}{\left[\begin{array}{c} A_{1}\cdots A_{K}\;B_{1}\cdots B_{K} \end{array}\right]}}}^{T},$$

where $A_{k}$ and $B_{k}$ are the inphase and quadrature components of the *k*-th harmonic, $f_{0}$ denotes the fundamental frequency of the harmonics. As $A_{k}$ and $B_{k}$ decrease at exponential rate as *k* increases [3], hence only the first *K* harmonics are considered in this paper. The estimate of the harmonics p(t) from y(t) can be derived by a least square (LS) estimation

(5) $$\text{minimize}\;\left(Y-XP\right){\left(Y-XP\right)}^{T}.$$

The fundamental frequency $f_{0}$ is obtained firstly by setting *K* = 1, followed with the estimation of *P*

(6) $$\hat{P}={\left(X^{T}X\right)}^{-1}X^{T}Y.$$

**Figure 5:**
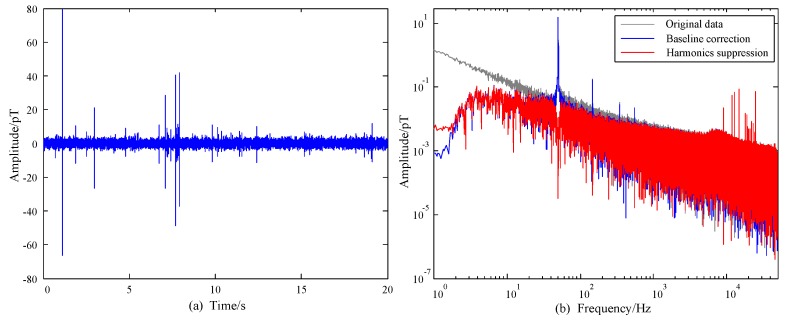
Processed data by the proposed method: (**a**) waveform after processing, and (**b**) comparison of Spectra.

When p(t) is obtained by (4) and (6), s(t) can be derived by subtracting p(t) from y(t) [24]. It can be found that p(t) largely depends on the estimate of $f_{0}$. However, $f_{0}$ is time-varying due to the drifting nature caused by generator and power plant design [3]. Consequently, the data should be divided into frames before LS estimation. A smaller length of the frame would lead to a better stationary characteristic of the data. While the performance of the LS estimation deteriorates as the number of samples decreases. Considering all these factors, the length of the frame Δt = 0.2 s and the number of harmonics *K* = 20 were chosen. The waveform and the corresponding spectra of the processed data are shown in Figure 5. It can be seen in Figure 5b that the background noise is quite low in comparison to measurements in urban area and there are only odd harmonics in the measured data. As a result, the distortion of the spectra can be diminished by designing X(t). The gray line denotes the spectra of the observed atmospheric noise before baseline correction. It can be seen that the low frequency components caused by baseline wandering are suppressed efficiently by wavelet denoising and the harmonics are further suppressed after LS estimation, which validates the effectiveness of the proposed method. To suppress the harmonics efficiently, the data were divided into frames before LS estimation. As a result, the low frequency part of the spectrum after harmonics suppression increases slightly in Figure 5b.

## 4. Statistical Analysis and Modeling for the Atmospheric Noise

**Figure 6:**
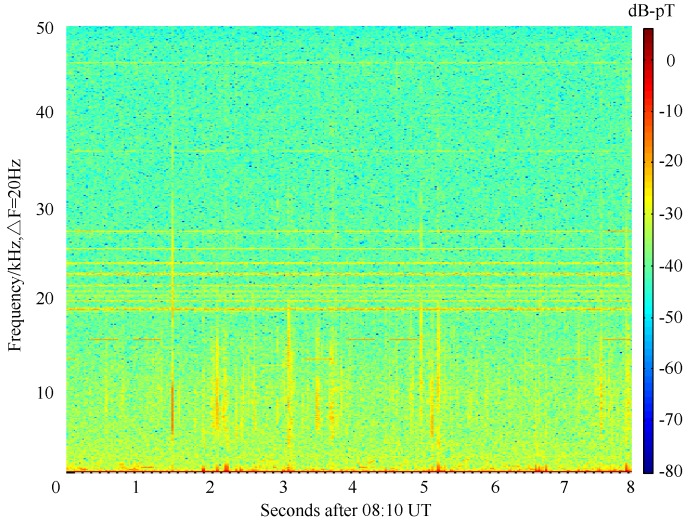
Spectrogram of the atmospheric noise.

Spectral analysis can only provide the global characteristics of the data without any time information. The data measured at 8:10 UT on 2nd March with a length of 8 s is shown in spectrogram form calculated by the function “spectrogram” in MATLAB in Figure 6. The data are divided into overlapping time bins, followed with a short-time Fourier transform performed on each time bin, between 0 and 50 kHz. The data bin size is 50 ms (i.e., ΔF = 20 Hz) with 50% overlapping. Longer bins have less bandwidth within each bin and thus higher resolution in spectra, but the time resolution is reduced, correspondingly. The amplitude of received signals in each frequency bin, and for each time bin, is indicated with the colorbar in Figure 6.

**Figure 7:**
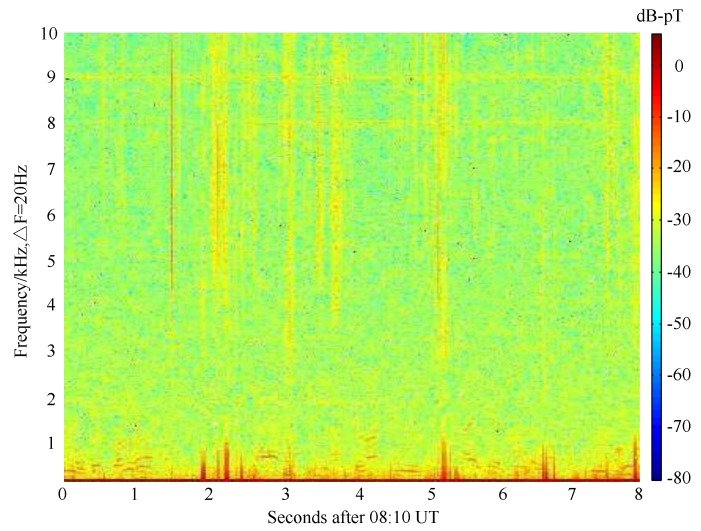
Spectrogram of the atmospheric noise lower than 10 kHz.

The thin vertical lines correspond to impulsive radio atmospherics, originated by lightning strikes, which can be observed at global distances from the receiver and are guided by the Earth-ionosphere waveguide. It can be observed that the magnetic field decreases as the frequency increases. This phenomenon is mainly due to the different attenuation of the electromagnetic wave propagated in the earth-ionosphere waveguide [25]. In order to get more details about the low-frequency noise characteristics, the spectrogram of the atmospheric noise lower than 10 kHz is given in Figure 7. It can be seen that the components of impulsive noise located in 1∼5 kHz are not significant and that may be the reason why 1.51 kHz and only its third harmonic are adopted in ELF/VLF communication using heating ionosphere in [7]. Moreover, there are many man-made interference mainly radiated by band limited VLF transmitter in Figure 6. The horizontal lines between 16 and 30 kHz correspond to constant minimum shift keying (MSK)-modulated VLF transmitters operated by various national navies for long-distance communication with naval vessels and submarines. The pulsed signals with a duration of 0.4 s between 10 and 15 kHz correspond to the so-called Alpha navigation system, a set of three VLF transmitters operated by Russia [13].

Limited by the bandwidth of the transmitter, existing works are mainly focused on the analysis and modeling for the narrowband ELF/VLF atmospheric noise. In [26], the atmospheric noise is regarded as symmetric *α*-stable (S*α*S) distribution and the bit error rate (BER) of Gaussian detectors for different digital modulation schemes are analyzed. Relevant analysis for the wideband ELF/VLF atmospheric noise is rarely reported, which limit the application of ELF/VLF communication by ionosphere heating. Mixed Gaussian distribution and Hall model are adopted to describe the pdf of the wideband ELF and VLF noise, respectively in [7]. However, comparisons with other widely used models are absent. Consequently, we perform statistical analysis and modeling for the wideband atmospheric noise to provide theoretical basis for the design of wideband communication system. According to the distribution of the carrier frequency, we choose 1.51 kHz and its third harmonic 4.53 kHz as the center frequency in ELF and VLF band, respectively. The collected data were filtered by a 16th-order Butterworth lowpass filter with a cutoff frequency of 5 kHz, followed by a factor of 10 downsampling. After this, the processed data were further filtered by narrowband channel filters with different bandwidthes, i.e., 50 Hz, 200 Hz and 400 Hz, respectively.

### 4.1. Normality Test for the Narrow Band Noise

We test the normality of the ELF/VLF data before statistical analysis. The data measured near 10:00 UT from 2nd to 7th March are used for the test, named Data1 to Data6, respectively. The data are divided into segments and Lilliefors test [27] is performed for them. As the transmission rates of ELF/VLF communication modulated by quadrature phase shift keying (QPSK) are 100, 400, and 800 bps in [7], the corresponding symbol rates are 50, 200, and 400 Baud/s. Consequently, the time length of the segments are chosen to be 20, 5.0, and 2.5 ms, respectively. The proportion of the normal distribution of the ELF and VLF atmospheric noise are shown in Table 1. It can be seen that the narrowband (20 ms) ELF/VLF data have very low proportion of normal distribution because impulses would be more likely to be included during a longer observation. As a result, the proportion of the normal distribution of the ELF/VLF data gets larger as the bandwidth increases.

**Figure 8:**
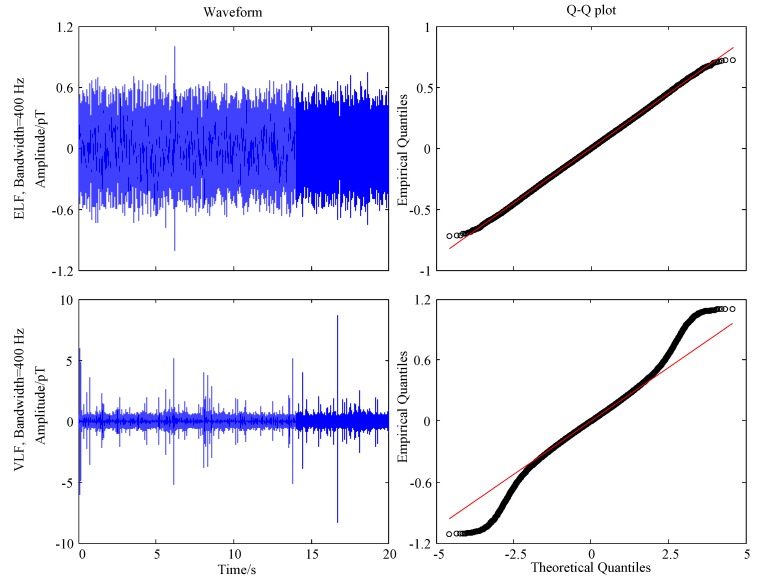
Waveform and the corresponding QQ -plot of the extremely low frequency (ELF) and very low frequency (VLF) noise. QQ-plot is performed for the noise processed by a hole puncher with a threshold of four times standard deviation.

In Table 1, we investigate the proportion of the normal distribution within one symbol period. However, we are also concerned about the noise distribution during a period of time in communication because it can give us a roughly statistical characteristic of the atmospheric noise. In order to illustrate an intuitive understanding of the noise distribution within a longer observation, the waveform of ELF/VLF noise with 400 Hz bandwidth from Data2 during 20 s is shown in Figure 8. It can be seen that ELF noise appears in an almost Gaussian distribution, accompanied with occasional impulses, while VLF noise exhibits more impulsive characteristics.

In statistics, a quantile-quantile (Q-Q) plot is a probability plot, which is a graphical method for comparing two probability distributions by plotting their quantiles against each other [28]. It has been widely used to compare a data set to a theoretical model to provide an assessment of “goodness of fit”. In order to investigate the distribution of low frequency atmospheric noise and the normal distribution intuitively, we perform the Q-Q plot graphically for ELF/VLF noise in Figure 8. To alleviate the adverse effect of the occasional impulses, the data are preprocessed by a hole puncher [29] with a threshold of 4*σ*, where *σ* is the standard deviation of the data. We may find that the processed ELF data mostly follow normal distribution and thus can be modeled by Gaussian noise. However, the VLF data deviate significantly from the normal distribution. It is consistent with the results shown in Table 1, in which the proportion of normal distribution of the ELF and VLF noise with 400 Hz bandwidth (2.5 ms) is 0.971 and 0.9, respectively. These conclusions are also consistent with that in [7].

### 4.2. Amplitude Probability Distribution of the Narrow Band Noise Envelope

The most measured and modeled statistic of low-frequency radio noise, next to absolute power level, is the first-order amplitude probability density (APD) [5]. It has been demonstrated that the bit error rate of some digital modulation can be well estimated by APD [30]. Other widely used statistical definitions that characterize the envelope of the noise A(t) are (1) the cumulative distribution function (CDF) $F_{A}\left(a\right)$, which is one minus the APD, (2) pdf $f_{A}\left(a\right)$ is the derivative of the CDF, and (3) the voltage deviation $V_{d}$ [5]. Traditional APD methods are based on the narrowband signal model. However, according to the Bedrosian’s product theorem in [31], the signal n(t)=A(t)cosθ(t) has the analytic form if the spectrum $S_{A}\left(f\right)=F\left\{A\left(t\right)\right\}$, where F is the Fourier operator, lies entirely in the region of $|f|<f_{0}$ and F{cosθ(t)} only exists outside of this region. Consequently, ELF/VLF signals n(t) considered here can be given as

(7) $$n\left(t\right)=n_{I}\left(t\right)\cos\left(2\pi ft\right)-n_{Q}\left(t\right)\sin\left(2\pi ft\right),$$

where, $n_{I}\left(t\right)$ is the in-phase component, $n_{Q}\left(t\right)$ is the quadrature component, and *f* is the center frequency. Both $n_{I}\left(t\right)$ and $n_{Q}\left(t\right)$ are real-valued lowpass signals and can be expressed by n(t) and its Hilbert transform $\hat{n}\left(t\right)$:

(8) $$n_{I}\left(t\right)=n\left(t\right)\cos\left(2\pi ft\right)+\hat{n}\left(t\right)\sin\left(2\pi ft\right),$$

(9) $$n_{Q}\left(t\right)=\hat{n}\left(t\right)\cos\left(2\pi ft\right)-n\left(t\right)\sin\left(2\pi ft\right).$$

Thus, the lowpass equivalent of n(t), denoted by $n_{l}\left(t\right)$, is given by

(10) $$n_{l}\left(t\right)=n_{I}\left(t\right)+jn_{Q}\left(t\right)=A\left(t\right)e^{j\theta (t)}.$$

It is known for a long time that the phase θ(t) of the atmospheric noise follows the uniform distribution over the angles −π to *π* [5]. As for the envelope A(t), there are several models used in recent literature to describe it, such as Hall model, Field and Lewenstein (F-L) model, Middleton’s Class A and Class B model, and *α*-stable distribution [32,33]. Considering the preliminary statistical results in [5], Hall model and S*α*S distribution are more suitable to describe the narrowband ELF/VLF noise. Consequently, only Rayleigh distribution, Hall model, and S*α*S distribution are considered in this paper.

The absolute value *r* of the complex random variable whose real and imaginary components follow independent identically distributed (i.i.d) Gaussian distribution with equal variance *σ* and zero mean is Rayleigh-distributed [34]. The pdf of the Rayleigh distribution is

(11) $$f_{\mathrm{Rayleigh}}\left(r\right)=\;\frac{r}{\sigma^{2}}e^{-\frac{r^{2}}{2\sigma^{2}}},r⩾0.$$

The Hall model is presented by Hall in 1966 and the envelope pdf is described as a two-parameter distribution [5]

(12) $$f_{\mathrm{Hall}}\left(r\right)=\;\left(m-1\right)\gamma^{m-1}\frac{r}{{\left(r^{2}+\gamma^{2}\right)}^{\left(m+1\right)/2}},r⩾0\;,$$

where *m* determines the impulsiveness of the noise and *γ* denotes the the scaling factor. It should be noted that the envelope has infinite variance for m⩽3, which is not physically possible.

While for the S*α*S noise, the closed form expression for its pdf is not available, except for special cases when the characteristic exponent *α* = 2 (Gaussian distribution) and *α* = 1 (Cauchy distribution). Instead, the S*α*S distribution is defined by its characteristic function

(13) $$\Phi_{X}\left(w\right)=e^{-\gamma {\left|w\right|}^{\alpha }},$$

where γ>0 is the dispersion, which measures the distribution’s spread. If the atmospheric noise follows S*α*S distribution, its envelope pdf is the Fourier-Bessel transform of $e^{-\gamma \rho^{\alpha }}$

(14) $$f_{S\alpha S}\left(r\right)=r\int_{0}^{\infty }\rho e^{-\gamma \rho^{\alpha }}J_{0}\left(r\rho \right)d\rho ,r⩾0,$$

where $J_{0}\left(\cdot \right)$ is the zero-th order Bessel function of the first kind. The distribution follows (14) is also called the heavy-tailed Rayleigh distribution [34]. This is due to the fact that for α=2 it is the Rayleigh distribution. While for α<2 it can describe the impulsive noise and has a thicker pdf tail compared to the Rayleigh distribution.

In order to compare the fitting performance of Rayleigh distribution, Hall model and S*α*S distribution, we should be able to estimate the model parameters from the observed data. Parameters of Rayleigh distribution and Hall model can be easily obtained by fitting the pdf with a LS algorithm. However, Equation (14) seems to be impossible to invert in order to estimate the required parameters. The pdf is approximated via numerical calculation and a lookup table method is adopted in [5]. However, this method need to store the lookup table and thus is not convenient to implement. In this paper, we adopt the fractional lower order moments method to estimate the parameters *α* and *γ*. The *p*-th order moment of the variable which follows the heavy-tailed Rayleigh distribution can be given as [34]

(15) $$E\left(r^{p}\right)=\frac{2^{p+1}\Gamma \left(\frac{p}{2}+1\right)}{\Gamma \left(\frac{p}{2}\right)}\frac{\gamma^{\frac{p}{\alpha }}\Gamma \left(-\frac{p}{\alpha }\right)}{\alpha },-2<p<-\frac{1}{2},$$

where $E\left(\cdot \right)$ is the expectation operator and $\Gamma \left(\cdot \right)$ is the Gamma function. Consequently, *α* can be estimated from the following formula

(16) $$\frac{E\left(r^{2p}\right)}{{\left[E\left(r^{p}\right)\right]}^{2}}=\frac{\Gamma \left(p+1\right)\Gamma^{2}\left(-\frac{p}{2}\right)}{2\Gamma \left(-p\right)\Gamma^{2}\left(\frac{p}{2}+1\right)}\frac{\alpha \Gamma \left(-\frac{2p}{\alpha }\right)}{\Gamma \left(-\frac{p}{\alpha }\right)},-1<p<-\frac{1}{2}.$$

Unfortunately, (16) is a highly nonlinear problem and cannot obtain a closed form solution for *α*. However, the Gamma function is well-behaved for the range of *p* and thus (16) can be solved by using the numerical optimization algorithms such as bisection [34]. Once *α* is obtained, *γ* can be derived from (15) as

(17) $$\gamma =\left(\frac{\Gamma \left(\frac{p}{2}\right)}{2^{p+1}\Gamma \left(\frac{p}{2}+1\right)}\frac{\alpha }{\Gamma \left(-\frac{p}{\alpha }\right)}\right),-1<p<-\frac{1}{2}.$$

As the envelope pdfs of the ELF/VLF noise exhibit heavy-tailed characteristic, mean square error (MSE) fails to provide a fair comparison between different models for the fitting. Thus, we adopt the mean-square log error (MSLE) [25] to evaluate the estimation performance. MSLE is defined as

(18) $$\mathrm{MSLE}=\int f_{A}\left(x\right){\left(\log_{10}\frac{f_{A}\left(x\right)}{{\hat{f}}_{A}\left(x\right)}\right)}^{2}dx,$$

where $f_{A}\left(x\right)$ is the data pdf and ${\hat{f}}_{A}\left(x\right)$ is the estimate by the model.

**Figure 9:**
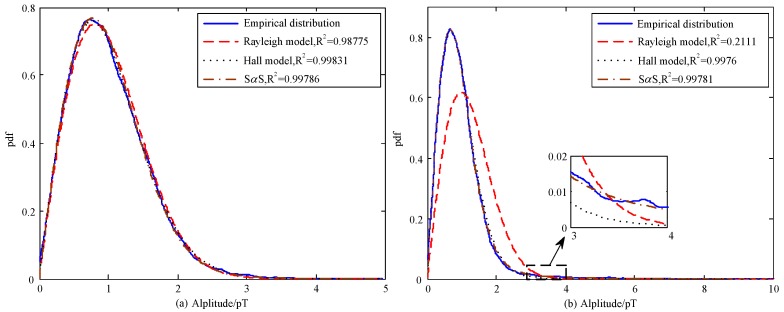
Comparisons of the fitting performance for (**a**) ELF atmospheric noise and (**b**) VLF atmospheric noise.

The envelope pdf fitting by Rayleigh distribution, Hall model, and S*α*S distribution for Data1 (Bandwidth is 400 Hz) are shown in Figure 9. As evidenced in Figure 9a, all the three models could be well fitted to the ELF atmospheric noise and the Hall model achieves the best performance. While for the VLF noise, the Rayleigh model behaves poor and S*α*S distribution outperforms others in the sense of goodness of fit. Moreover, S*α*S describes the heavy-tail characteristic of the VLF noise more precisely in the zoom area. In order to get a quantitative analysis of the fitting performance, the envelope *A* of all the 42 sets of data are normalized such that the average envelope values E[A]=1. The parameters of Data1 with different frequency bands estimated by the three models are revealed in Table 2. It can be further validated that VLF noise contains more impulsive components and thus cannot be characterized by Gaussian model. The S*α*S distribution achieves smaller MLSE than the other two models because it can get more accurate fitting for the heavy tail. For the ELF noise, the MLSE of the Rayleigh model decreases significantly because ELF noise appears mostly Gaussian. All of the three models achieve good fitting performance in the sense of MLSE.

Statistical analysis for all the 42 data sets show that, about 61% of the ELF noise are more suitable to be described by Hall model. Values of impulse index *m* range from 20 to 270 and values of *γ* range from 3 to 13. When investigating the VLF noise, about 75% of the noise follow S*α*S distribution and thus S*α*S is most suitable. Thereby, *α* is usually in the range of 1.8 to 1.96, although values as low as 1.73 and as high as 2 are also found. The corresponding *γ* is mainly distributed from 0.29 to 0.32. As the increase of bandwidth, more noise radiated from the lightings all over the world will be observed and thus the noise amplitude tend to rise. While the impulsive characteristics turn to weak as the increase of noise source due to the central limit theorem (CLT) [35]. It should be noted that *α* fitted by S*α*S distribution is mainly located in the range of 1.9 to 2 for ELF noise, indicating that the impulsive characteristic is very weak and can be simplified to Gaussian noise when a hole puncher is performed for the occasional impulses. Consequently, from the qualitative comparison in Figure 9 and quantitative comparisons in Table 2, we can derive the conclusion that the ELF/VLF noise considered in this paper follows Gaussian distribution, with occasional impulses from lighting strikes. For simplicity, ELF noise can be regarded as Gaussian noise after preprocessing by a hole puncher operator for impulses. While for VLF noise, S*α*S distribution is optimal to describe the data. The simulation results are also consistent with the conclusion in [7].

## 5. Conclusions

A low-temperature SQUID receiver was adopted to observe the ELF/VLF atmospheric noise for the first time in this paper. Symmetric wavelet decomposition and LS estimation were adopted to preprocess the data to suppress baseline wandering caused by geomagnetic activity and harmonics radiated from power line. Analysis for the temporal atmospheric noise show that the ELF/VLF noise have non-Gaussian characteristic, especially for the narrowband noise. Modeling of the ELF/VLF noise provides the insight that, Hall model is the optimal choice to depict the APD of the ELF noise, while S*α*S can fit the heavy-tail characteristic of the VLF noise more precisely. As *α* fitted by S*α*S distribution are mainly in the range of 1.9 to 2, the ELF noise can be regarded as Gaussian distribution for simplicity after preprocessing the impulses by a hole puncher.

The initial results of this study may allow for the modeling of wideband ELF/VLF communication performances, given the improved knowledge of the characteristics of the natural background noise for the design of an optimal SQUID based receiver. The parameters derived in this work can also be used as input parameters when trying to simulate low-frequency wideband communication. Longer observation time and more receivers in different districts will be adopted in the future to derive a more general conclusion.

## Figures and Tables

**Table 1:** The proportion of the normal distribution of the atmospheric noise at different frequency bands.

Frequency Band	Length	Data1	Data2	Data3	Data4	Data5	Data6
ELF	20 ms	0.42	0.361	0.377	0.395	0.382	0.386
	5 ms	0.872	0.866	0.866	0.868	0.868	0.864
	2.5 ms	0.974	0.971	0.973	0.973	0.974	0.971
VLF	20 ms	0.309	0.357	0.363	0.348	0.324	0.324
	5 ms	0.747	0.753	0.756	0.789	0.771	0.761
	2.5 ms	0.892	0.9	0.912	0.923	0.921	0.914

**Table 2:** Fitting performance for the ELF noise by the two models.

Noise	Model	50 Hz		200 Hz		400 Hz	
		Parameters	MSLE	Parameters	MSLE	Parameters	MSLE
ELF	Rayleigh	*σ* = 0.823	8.59 $\times \;10^{-4}$	*σ* = 0.809	1.82 $\times \;10^{-4}$	*σ* = 0.807	2.04 $\times \;10^{-4}$
	Hall	*m* = 19.68	2.15 $\times \;10^{-4}$	*m* = 40.43	4.67 $\times \;10^{-5}$	*m* = 30.18	4.65 $\times \;10^{-4}$
		*γ* = 3.349		*γ* = 4.939		*γ* = 4.212	
	S*α*S	*α* = 1.833	5.89 $\times \;10^{-4}$	*α* = 1.945	6.75 $\times \;10^{-5}$	*α* = 1.96	6.55 $\times \;10^{-5}$
		*γ* = 0.317		*γ* = 0.316		*γ* = 0.316	
VLF	Rayleigh	*σ* = 0.91	1.32 $\times \;10^{-2}$	*σ* = 0.959	1.81 $\times \;10^{-2}$	*σ* = 0.978	1.86 $\times \;10^{-2}$
	Hall	*m* = 8.303	2.09 $\times \;10^{-4}$	*m* = 12.76	3.48 $\times \;10^{-4}$	*m* = 15.68	3.41 $\times \;10^{-4}$
		*γ* = 1.823		*γ* = 2.356		*γ* = 2.681	
	S*α*S	*α* = 1.736	1.04 $\times \;10^{-4}$	*α* = 1.776	8.04 $\times \;10^{-5}$	*α* = 1.803	6.15 $\times \;10^{-5}$
		*γ* = 0.297		*γ* = 0.293		*γ* = 0.292	
